# Bees of the Mediterranean basin: biodiversity insights from specimens in the IMBE collection (Marseille, France)

**DOI:** 10.3897/BDJ.12.e141734

**Published:** 2024-12-27

**Authors:** Louhane Schneider, Charlène Lossouarn, Benoît Geslin, Coline C. Jaworski, Lucie Schurr, Lise Ropars, Claire Bouchot, Marie Zakardjian, Floriane Flacher, Matthieu Aubert, David Genoud, Éric Dufrêne, Vincent Leclercq, Gabriel Nève

**Affiliations:** 1 Aix Marseille University, Marseille, France Aix Marseille University Marseille France; 2 IMBE, Marseille, France IMBE Marseille France; 3 Avignon University, Avignon, France Avignon University Avignon France; 4 CNRS, Marseille, France CNRS Marseille France; 5 IRD, Marseille, France IRD Marseille France; 6 Rennes University, Rennes, France Rennes University Rennes France; 7 Institute of Ecology and Environmental Sciences, Sorbonne Université, Paris, France Institute of Ecology and Environmental Sciences, Sorbonne Université Paris France; 8 MNHN, Paris, France MNHN Paris France; 9 Observatoire des Abeilles, Flines-lez-Raches, France Observatoire des Abeilles Flines-lez-Raches France; 10 Independant researcher, Ambazac, France Independant researcher Ambazac France

**Keywords:** Hymenoptera, Apoidea, distribution, France, Andrenidae, Apidae, Colletidae, Halictidae, Megachilidae, Melittidae

## Abstract

**Background:**

The spectacular decline in pollinators and their prominent role in pollination of natural and cultivated plants has stimulated research on pollinating insects. Over the last ten years, much ecological research has been carried out on bees, often generating a large volume of specimens and increasing the importance of entomological collections. Here, we present the bee collection of the IMBE laboratory (Marseille, France) after ten years of study of plant-pollinator networks.

**New information:**

We provide distribution data on 2181 specimens belonging to 246 species of bees, mainly from the Mediterranean Region of France. One of the recorded species, *Lasioglossumsoror*, is classified as "endangered" at the European level, while 68 of the recorded species are currently Data Deficient according to the 2014 Red List of European bees. This dataset contributes to the broader effort to enhance the knowledge of French bee diversity. It aligns with the objectives of the French Pollinator Plan and supports the development of a national Red List. In this context, information about the distribution of wild bees from the Mediterranean Region, which harbours the highest species diversity in mainland France, are of particular importance.

## Introduction

Dramatic declines in many pollinator populations have been documented worldwide (Raven and Wagner 2021) raising concerns about the maintenance of their species through time and space and the sustainability of the ecosystem services they provide. In this context, accurate data on pollinator distribution is crucial to document their current status. Amongst these, specimens preserved in natural history collections are reliable pieces of the puzzle that describes ecological communities, habitats and ecosystem health (Raven and Miller 2020). Opening these collections and making them accessible through digitisation and publication is, therefore, of paramount importance for generating science-driven conservation strategies.

Over the past decades, researchers at the Mediterranean Institute of marine and continental Biodiversity and Ecology (IMBE) in Marseille have been investigating relationships between pollinators and plants across a variety of habitats in France, with a focus on the Bouches-du-Rhône Department (Schurr et al. 2019, Ropars et al. 2020a, Ropars et al. 2020b, Schurr et al. 2021, Schurr et al. 2022, Carisio et al. 2022, Jaworski et al. 2022, Badiane et al. 2024). Several projects have been undertaken by the Institute, including field courses on bee identification (Geslin et al. 2018) and field campaigns in the Calanques National Park and the City of Marseille. These campaigns used standardised sampling protocols such as netting, pan-trapping and trap-nesting (insect hotels, Geslin et al. (2020)). This work generated the collection of many specimens that have been kept in the IMBE since 2015. Additionally, some specimens were sent to enrich the collection from some of the renowned professional taxonomists of France (namely M. Aubert, E. Dufrêne and D. Genoud). Each of the specimens collected has been meticulously pinned, labelled and was frozen at least twice a year to avoid the emergence of pests.

This paper aims to publish the raw data of bee specimens currently housed in the IMBE collections. These include specimens collected during studies, as well as those donated to the Institute, with the exception of specimens provided to professional taxonomists, lost, destroyed during preparation or used for DNA sequencing, representing a small fraction of the collection. This dataset serves as a valuable resource for advancing our understanding of the Mediterranean Basin's bee fauna and supports ongoing efforts to develop national checklists and Red Lists.

## General description

### Purpose

The dataset presented here includes information on a total of 2,181 bee specimens, collected between 2003 and 2023, all identified to the species level, along with detailed information on the date and location of each capture. This dataset includes some Mediterranean species for which few published records are available in national (inpn.mnhn.fr) and international (www.gbif.org) databases.

### Additional information

The project aims to compile all recorded data on Apoidea, collected during field campaigns and from donations over the past 10 years, as well as opportunistic captures sent by entomologists to the IMBE laboratory. A total of three scientific field campaigns were conducted in the protected area of the Calanques National Park (Geslin et al. 2018, Ropars et al. 2018, Schurr et al. 2019), two in the urban parks of Marseille (Geslin et al. 2020, Badiane et al. 2024), one in humid area (Solère et al. 2022) and three within the Alpes-de-Haute-Provence Regional Park (Schurr et al. 2021, Carisio et al. 2022, Schurr et al. 2022). A few additional specimens were gathered elsewhere in France, Spain and Italy and sent to the IMBE.

## Sampling methods

### Sampling description

For each specimen, the following information was retrieved: species, date and location of capture, sex and collector, as well as any relevant ecological data, such as the plant on which the specimen was captured. Specimens were captured with an entomological net or with coloured pan traps. In addition to the data on the original labels, each specimen was assigned a unique identification code written on an added label.

### Quality control

The specimens were identified by Mathieu Aubert, Claire Bouchot, Eric Dufrêne, Benoît Geslin, Vincent Leclercq, Lise Ropars, Lucie Schurr, Erwin Scheuchl, Benoît Martha, Emile Gigandet, Gilles Mahé, Yvan Brugerolles, Gérard Le Goff and Nicolas J. Vereecken. The taxonomy was checked to be compatible with the French TAXREF v.17,0 from the Museum national d’Histoire naturelle (Paris) data base (Gargominy et al. 2021) and with the latest checklist of the bees of the French fauna (Ropars et al. in press). In case of name change due to taxonomic update or identification correction, the original name given to the specimen was retained in the 'previousIdentifications' column.

### Step description

All data on the 2181 identified specimens currently in the IMBE collection were input in a table format. Latitude and longitude coordinates of each capture location were obtained either directly from the label or inferred from the location description. In the second case, coordinates were retrieved using the geoportail.gouv.fr website. Coordinates originally in Lambert93 format and in degrees minutes seconds were transformed to standard GPS format (latitude and longitude in decimal degrees). The locations were then verified using the https://www.geoportail.gouv.fr/ website. Coordinates uncertainty (‘coordinateUncertaintyInMeters’ column) was set to at least 100 m, depending on the level of detail provided on the capture location on the specimen label. All formats follow GBIF Darwin Core specification, to ensure interoperability with other international databases.

For 32 specimens, only identification to the genus level was possible. This is indicated by a value of 0 in the ‘identificationVerificationStatus’ column, with relevant additional details provided in the ‘identificationRemarks’ column. Species groups were indicated with ‘gr.’ in the ‘identificationQualifier’ column, such as for the *Bombusterrestris* species group, which requires genetic data for accurate species-level identification (Murray et al. 2008). ‘cf.’ was used to indicate an approximate identification. This was the case of some *Hylaeus* specimens which could only be assigned to a couple of species: *H.pictipes* or *H.taeniolatus*.

All data were entered in CSV format, with the fields separated by tabs and encoded in UTF-8, thus following the protocol compatible with the GBIF database (Global Biodiversity Information Facility, https://www.gbif.org/fr/) as used in previously published datasets (Meunier et al. 2023, Nève et al. 2024, Ollivier et al. 2024).

## Geographic coverage

### Description

Most of the specimens come from France (2178), one is from Spain and two are from Italy.

**Figure 1.**
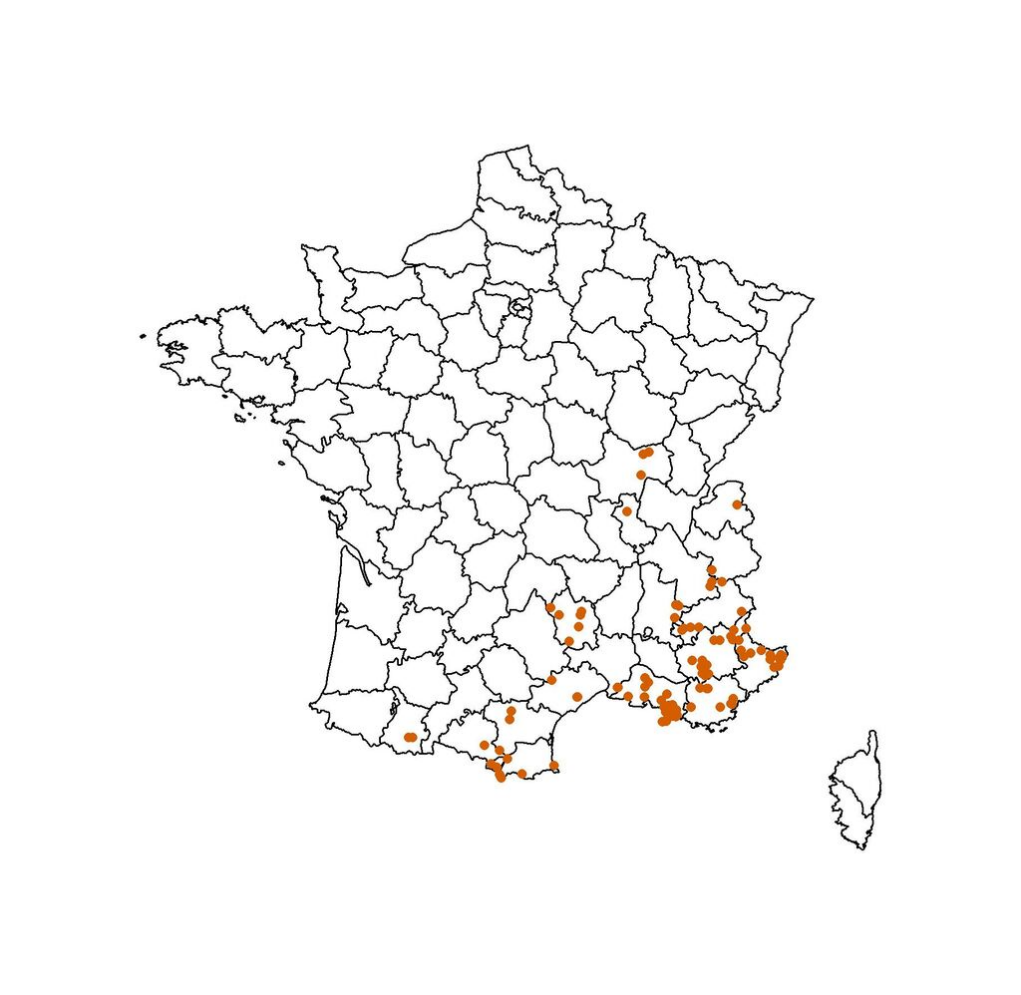
Distribution of Anthophila specimens in the IMBE collection. Specimens from the Departments of Yvelines (n = 13), Seine-et-Marne (n = 4), Manche (n = 2) and Essonne (n = 8) are omitted. One specimen from Spain and two from Italy are omitted.

In France, 93% the data come from the Bouches-du-Rhône Department, with some from Var, Alpes-Maritimes and Alpes-de-Haute-Provence (Fig. 1; Suppl. material 1).

### Coordinates

39.30 and 49.33 Latitude; -1.28 and 9.20 Longitude.

## Taxonomic coverage

### Description

The dataset covers 246 species of Apoidea belonging to the six bee families found in France: Andrenidae, Apidae, Colletidae, Halictidae, Megachilidae and Melittidae (Tables 1, 2).

## Temporal coverage

**Data range:** 2003-3-20 – 2023-9-01.

### Notes

**Figure 2.**
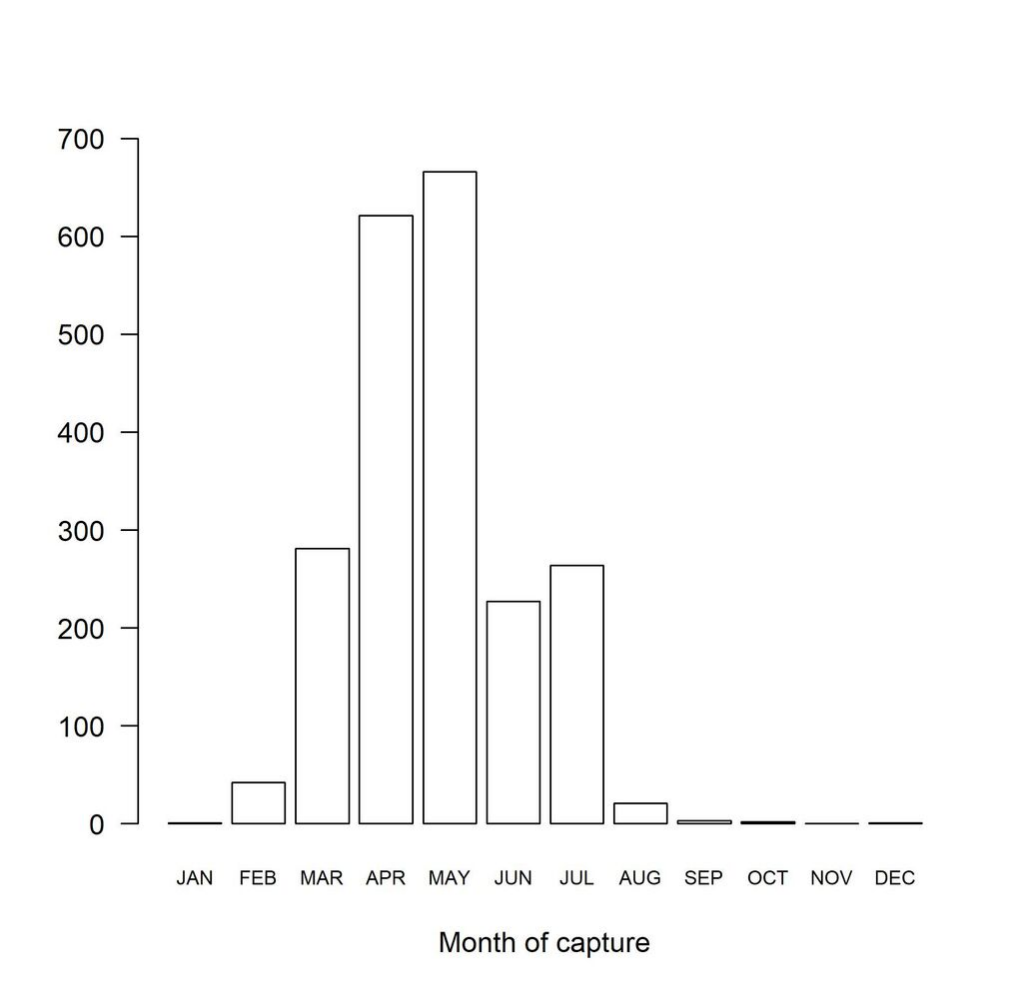
Within year distribution of bees in the IMBE collection.

The specimens were captured from 2003 to 2023. Twenty specimens were collected at an unknown date. Most of the specimens were captured in April or May (the time of the year in which bees are the most abundant in this region), totalling 60% of the dated captures (Fig. 2).

## Collection data

### Collection name

IMBE Apoidea collection (Hymenoptera)

### Collection identifier

IMBE-H

### Specimen preservation method

Dried and pinned specimens

### Curatorial unit

IMBE, contact: Gabriel Nève (email: gabriel.neve@imbe.fr)

## Usage licence

### Usage licence

Creative Commons Public Domain Waiver (CC-Zero)

### IP rights notes

This work is licensed under a Creative Commons Attribution (CC-BY) 4.0 Licence. All work derived from the present study should cite it appropriately.

## Data resources

### Data package title

IMBE Bee collection (Hymenoptera)

### Resource link

https://doi.org/10.5281/zenodo.13936315

### Alternative identifiers

https://doi.org/10.5281/zenodo.14356371

### Number of data sets

1

### Data set 1.

#### Data set name

IMBE Apoidea collection (Hymenoptera)

#### Data format

CSV (tab delimited values)

#### Character set

IMBE_beeColl_v02.csv

#### Data format version

Darwin core, so that it may be transferred later into GBIF.

#### Description

The dataset includes data on 2181 specimens of Apoidea collected or received by researchers at IMBE, in GBIF compatible format.

**Data set 1.** 

Column label	Column description
occurrenceID	Unique specimen identifier : H (for Hymenoptera) and four digits.
catalogNumber	Insitution code (i.e. IMBE) followed by individual occurenceID. Each specimen bears a label with this identifier.
basisOfRecord	The specific nature of the data record (i.e. PreservedSpecimen).
eventDate	Event date in the format YYYY-MM-DD if the capture date is known to the date or YYYY if only the year is known. If time of capture is known, then format is YYYYMM-DDTHH:MM, with HH:MM the local time.
year	Year of capture.
month	Month of capture if known,
day	Day of capture if known,
verbatimEventDate	Date of capture, as mentioned on the label,
scientificName	Lowest taxonomic rank possible, usually the species name, sometimes the subspecies, with author and year.
identificationQualifier	In case the identification could be given only to a species group, 'cf.' was input.
identificationRemarks	Any comment on the identification of the specimen, with list of possible species.
kingdom	Kingdom name (i.e. Animalia).
phylum	Phylum name (i.e. Arthropoda).
class	Class name (i.e. Insecta).
order	Order name (i.e. Hymenoptera).
family	Family name.
genus	Genus name.
specificEpithet	Species epithet of the scientificName.
infraspecificEpithet	Subspecific epithet, if any is relevant.
scientificNameAuthorship	Name of the scientist who described the species and year of description publication.
sex	Male (M) or female (F).
caste	Queen or worker if known.
taxonRank	Taxonomic rank of the most specific name in the scientificName.
identifiedBy	Name of the entomologist who identified the specimen.
dateIdentified	Year of identification.
identificationVerificationStatus	Whether (coded 1) or not (coded 0) the identification is reliable to species level.
previousIdentifications	Species name originally given on the specimen label.
country	Country of capture.
countryCode	Two letter country code of the specimen capture location.
stateProvince	French departmental administrative division. In the case of non-French data, any relevant country administrative subdivision.
locality	Location of capture, usually the municipality.
verbatimLocality	Any geographical indication on the label.
occurrenceRemarks	Any ecological data or comment on the label, including method of capture if known.
decimalLatitude	Geographic latitude (in decimal degrees) of the capture location.
decimalLongitude	Geographic longitude (in decimal degrees) of the capture location.
geodeticDatum	System and set of reference points upon which the geographic coordinates are based (i.e. WGS 84)
coordinateUncertaintyInMeters	Uncertainty in coordinates, in meters
minimumElevationInMeters	Lower limit of the range of altitudes indicated on the label or in the associated reference
maximumElevationInMeters	Higher limit of the range of altitude indicated on the label or in the associated reference.
georeferencedBy	Identity of the person who added the latitude and longitude data, i.e. either the original recorder or Nève, Gabriel.
georeferenceProtocol	How the georeference was computed, i.e. either from georeference web sites or from label data (verbatimLocality).
georeferenceSources	Georeference code was inferred from geoportail.gouv.fr, French ING maps or GoogleEarthPro.
georeferencedDate	Georeference work was either performed at the time of recording or in 2024.
recordedBy	Name of collector (i.e. legit information).
otherCatalogNumbers	Any other code the specimen may have, usually according to the study protocol during which it was captured.
institutionCode	Institution where the specimen is held (i.e. IMBE, Marseille).
organismQuantity	Number of individuals bearing the same label (usually 1).
organismQuantityType	Individuals.
language	The dataset is mainly written in French, apart from column headings, which are in English.
associatedReferences	Any reference citing the relevant specimen.

## Additional information

### General discussion

At a time of global pollinator decline, this study highlights once again the French Mediterranean Region as one of the main hotspots for wild bee species (Orr et al. 2021, Reverté et al. 2023). The collection holds about a quarter of the 980 species that thrive in mainland France (Ropars et al. in press) and the data provided here will contribute to improve knowledge about French bee species.

The collected specimens listed here, beyond enhancing our understanding of wild bees as previously mentioned, are also used as a reference collection and for the training of entomologists to ensure the accurate identification of future specimens. Moreover, such a collection acts as a 'memory of nature's diversity,' an exceptional resource available to scientists worldwide. Preserving these specimens provides tangible evidence of biodiversity over time, offering a unique opportunity to monitor its evolution in the long term — a critical aspect in the face of the current widespread biodiversity decline and the current major global changes.

One species in the IMBE collection is classified as 'endangered' by the IUCN at the European level (Nieto et al. 2014). This species, *Lasioglossumsoror*, was recorded at nine different localities in the Calanques National Park in 2018. Our data suggest that this species is locally widespread and may have been overlooked in other studies as all but two of the 19 specimens in the IMBE collection were collected in coloured pan traps.

Six species in the IMBE collection are classified as “Near Threatened” by the IUCN at the European level (Nieto et al. 2014): *Andrenaovatula*, *Colletesalbomaculatus*, *Lasioglossumprasinum*, *Lasioglossumpygmaeum*, *Lasioglossumsexnotatum* and *Dasypodaargentata*. In addition, *D.argentata* is also generally recognised as a rare species. The identification of these species is crucial for conservation efforts, as it guides habitat management and protection initiatives. Understanding the status of near-threatened species helps preserve biodiversity and maintain essential ecosystem services provided by pollinators, while raising public awareness and support for bee conservation initiatives. Finally, a total of 68 species recorded in the IMBE collection are currently classified as 'Data Deficient'. This status underscores significant gaps in our understanding of these species' biology, distribution and conservation needs. It highlights the critical importance of maintaining and enhancing monitoring efforts to gather the data necessary for informed conservation decisions. Ensuring the availability of updated and comprehensive information on these species is essential to address potential threats and support their long-term survival.

This study is part of current national effort to document the distribution of pollinators in France. Many initiatives, for example, French National Pollinator Plan, Regional Initiatives ("Plan Régional d'Action") and the French Checklist (Ropars et al. in press) are currently assessing the status of wild bees species in France and this topical issue is part of the research programme. By contributing valuable data to the forthcoming French Red List of bees, we aim to foster a greater awareness of the ecological significance of these species. As the IMBE continues its dedicated research, we anticipate that future studies will further enrich our knowledge and help shape effective conservation strategies, ensuring that these vital pollinators thrive for generations to come.

### Abbreviation used throughout

IMBE: Institut Méditerranen de Biodiversité et d’Ecologie marine et continentale (Marseille, France)

## Supplementary Material

B876D59A-D6CC-5E70-9529-9FF55246FF32 10.3897/BDJ.12.e141734.suppl1 Supplementary material 1 Numbers of specimens per French Department Data type geographic coverage of bee data in France Brief description Numbers of bee specimens per French Department in the IMBE collection (Marseille, France). Encoding is UTF-8.
Two specimens from Italy (Sardinia) and one from Spain (Catalonia) are exluded. File: oo_1201694.csv https://binary.pensoft.net/file/1201694 Gabriel Nève

## Figures and Tables

**Table 1.** Numbers of species and specimens per family in the IMBE Anthophila collection.

Family	Number of species	Number of specimens
Andrenidae	47	301
Apidae	73	649
Colletidae	19	80
Halictidae	44	318
Megachilidae	61	831
Melittidae	2	2
**Total**	**246**	**2181**

**Table 2.** Number of specimens per species in the IMBE Anthophila collection. Nomenclature follows Ropars et al. (in press). European Red List status follows Nieto et al. (2014). DD: Data Deficient, EN: Endangered, LC: Low Concern, NT: Near Threatened. *Megachilescupturalis* was not assessed in the Red List as it is an introduced species (Le Féon et al. 2018).

**Family**	**Species**	**European Red List status**	**Number of specimens**
Andrenidae	*Andrenaagilissima* (Scopoli, 1770)	DD	2
Andrenidae	*Andrenaangustior* Kirby, 1802	DD	1
Andrenidae	*Andrenabicolor* Fabricius, 1775	LC	9
Andrenidae	*Andrenabimaculata* Kirby, 1802	DD	2
Andrenidae	*Andrenachrysosceles* (Kirby, 1802)	DD	1
Andrenidae	*Andrenacineraria* Linnaeus, 1758	LC	2
Andrenidae	*Andrenacinerea* Brullé, 1832	DD	1
Andrenidae	*Andrenacombinata* (Christ, 1791)	DD	14
Andrenidae	*Andrenadorsata* (Kirby, 1802)	DD	3
Andrenidae	*Andrenafabrella* Pérez, 1903	DD	10
Andrenidae	*Andrenafalsifica* Perkins, 1915	DD	1
Andrenidae	*Andrenaflavipes* Panzer, 1799	LC	3
Andrenidae	*Andrenafulva* (Müller, 1766)	DD	1
Andrenidae	*Andrenafuscipes* (Kirby, 1802)	DD	2
Andrenidae	*Andrenahaemorrhoa* (Fabricius, 1781)	LC	2
Andrenidae	*Andrenahesperia* Smith, 1853	LC	17
Andrenidae	*Andrenahumilis* Imhoff, 1832	DD	1
Andrenidae	*Andrenalabiata* Fabricius, 1781	DD	1
Andrenidae	*Andrenalagopus* Latreille, 1809	LC	10
Andrenidae	*Andrenalimbata* Eversmann, 1852	DD	1
Andrenidae	*Andrenamarginata* Fabricius, 1776	DD	1
Andrenidae	*Andrenaminutula* Kirby, 1802	DD	2
Andrenidae	*Andrenamorio* Brullé, 1832	DD	1
Andrenidae	*Andrenanigroaenea* Kirby, 1802	LC	58
Andrenidae	*Andrenanitida* Müller, 1776	LC	1
Andrenidae	*Andrenaniveata* Friese, 1887	DD	28
Andrenidae	*Andrenaovatula* Kirby, 1802	NT	3
Andrenidae	*Andrenapandellei* Pérez, 1895	LC	2
Andrenidae	*Andrenapilipes* Fabricius, 1781	LC	1
Andrenidae	*Andrenapolita* Smith, 1847	LC	1
Andrenidae	*Andrenapraecox* (Scopoli, 1763)	LC	1
Andrenidae	*Andrenapusilla* Pérez, 1903	DD	2
Andrenidae	*Andrenarhenana* Stöckhert in Schmiedeknecht, 1930	DD	10
Andrenidae	*Andrenarufula* Schmiedeknecht, 1883	LC	1
Andrenidae	*Andrenasenecionis* Pérez, 1895	LC	8
Andrenidae	*Andrenasimilis* Smith, 1849	DD	19
Andrenidae	*Andrenasimillima* Smith, 1851	LC	1
Andrenidae	*Andrenasimontornyella* Noskiewicz, 1939	LC	1
Andrenidae	*Andrenaspreta* Pérez, 1895	DD	2
Andrenidae	*Andrenasubopaca* Nylander, 1848	LC	1
Andrenidae	*Andrenatenuistriata* Pérez, 1895	LC	3
Andrenidae	*Andrenatruncatilabris* Morawitz, 1877	DD	1
Andrenidae	*Andrenavaga* Panzer, 1799	LC	1
Andrenidae	*Andrenavetula* Lepeletier, 1841	LC	1
Andrenidae	*Andrenavillipes* Pérez, 1895	LC	4
Andrenidae	*Andrenavulpecula* Kriechbaumer, 1873	DD	33
Andrenidae	*Panurgusdentipes* Latreille, 1811	LC	30
Apidae	*Amegillaalbigena* (Lepeletier, 1841)	LC	3
Apidae	*Amegillagarrula* (Rossi, 1790)	LC	1
Apidae	*Ammobatoidesscriptus* (Gerstäcker, 1869)	DD	1
Apidae	*Anthophoraaestivalis* (Panzer, 1801)	LC	5
Apidae	*Anthophoraaffinis* Lepeletier, 1841	DD	11
Apidae	*Anthophoraatriceps* Pérez, 1879	DD	1
Apidae	*Anthophorabimaculata* (Panzer, 1798)	LC	7
Apidae	*Anthophoracrassipes* Lepeletier, 1841	DD	1
Apidae	*Anthophoracrinipes* Smith, 1854	DD	13
Apidae	*Anthophoradispar* Lepeletier, 1841	LC	64
Apidae	*Anthophorafemorata* (Olivier, 1789)	DD	4
Apidae	*Anthophoramucida* Gribodo, 1873	DD	8
Apidae	*Anthophoraplumipes* (Pallas, 1772)	LC	73
Apidae	*Apismellifera* Linnaeus, 1758	DD	90
Apidae	*Bombushortorum* (Linnaeus, 1761)	LC	2
Apidae	*Bombushumilis* Illiger, 1806	LC	5
Apidae	*Bombuslucorum* (Linnaeus, 1761)	LC	6
Apidae	*Bombusmastrucatus* Gerstäcker, 1869	LC	2
Apidae	*Bombusmesomelas* Gerstäcker, 1869	LC	5
Apidae	*Bombusmonticola* Smith, 1849	LC	4
Apidae	*Bombuspascuorum* (Scopoli, 1763)	LC	33
Apidae	*Bombuspratorum* Linnaeus, 1758	LC	4
Apidae	*Bombusruderarius* (Müller, 1776)	LC	2
Apidae	*Bombussichelii* Radoszkowski, 1859	LC	1
Apidae	*Bombussoroeensis* (Fabricius, 1777)	LC	2
Apidae	*Bombussylvarum* (Linnaeus, 1761)	LC	2
Apidae	*Bombusterrestris* Linnaeus, 1758	LC	47
Apidae	*Ceratinachalcites* Germar, 1839	LC	2
Apidae	*Ceratinacucurbitina* Rossi, 1792	LC	13
Apidae	*Ceratinacyanea* (Kirby, 1802)	LC	12
Apidae	*Ceratinadallatorreana* Friese, 1896	LC	1
Apidae	*Ceratinadentiventris* Gerstäcker, 1869	LC	4
Apidae	*Ceratinagravidula* Gerstäcker, 1869	LC	2
Apidae	*Ceratinanigrolabiata* Friese, 1896	LC	6
Apidae	*Epeolusjulliani* Pérez, 1884	LC	1
Apidae	*Euceracaspica* Morawitz, 1873	LC	89
Apidae	*Euceraclypeata* Erichson, 1835	LC	3
Apidae	*Eucerahispana* Lepeletier, 1841	DD	8
Apidae	*Eucerainterrupta* Baer, 1850	LC	1
Apidae	*Euceralongicornis* (Linnaeus, 1758)	LC	1
Apidae	*Euceranigrescens* Pérez, 1880	LC	4
Apidae	*Euceranigrifacies* Lepeletier, 1841	LC	5
Apidae	*Euceranigrilabris* Lepeletier, 1841	DD	1
Apidae	*Eucerarufa* (Lepeletier, 1841)	DD	1
Apidae	*Eucerataurica* Morawitz, 1871	DD	2
Apidae	*Euceravulpes* Brullé, 1832	DD	1
Apidae	*Melectaalbifrons* (Förster, 1771)	LC	1
Apidae	*Melectaitalica* Radoszkowski, 1876	DD	2
Apidae	*Nomadabeaumonti* Schwarz, 1967	LC	3
Apidae	*Nomadabluethgeni* Stoeckhert, 1943	LC	1
Apidae	*Nomadadiscedens* Pérez, 1884	LC	9
Apidae	*Nomadadiscrepans* Schmiedeknecht, 1882	LC	2
Apidae	*Nomadadistinguenda* Morawitz, 1873	LC	1
Apidae	*Nomadafabriciana* (Linnaeus, 1767)	LC	1
Apidae	*Nomadafacilis* Schwarz, 1967	LC	1
Apidae	*Nomadafemoralis* Morawitz, 1869	LC	1
Apidae	*Nomadaflavoguttata* (Kirby, 1802)	LC	11
Apidae	*Nomadafulvicornis* Fabricius, 1792	LC	1
Apidae	*Nomadafurvoides* Stöckhert, 1944	DD	1
Apidae	*Nomadagoodeniana* (Kirby, 1802)	LC	1
Apidae	*Nomadaintegra* Brullé, 1832	LC	3
Apidae	*Nomadamaculicornis* Pérez, 1884	DD	4
Apidae	*Nomadamarshamella* (Kirby, 1802)	LC	1
Apidae	*Nomadamocsaryi* Schmiedeknecht, 1882	DD	1
Apidae	*Nomadapanurgina* Morawitz, 1868	LC	4
Apidae	*Nomadasheppardana* (Kirby, 1802)	LC	20
Apidae	*Nomadasuccincta* Panzer, 1798	LC	10
Apidae	*Tetraloniafulvescens* (Giraud, 1863)	DD	1
Apidae	*Tetraloniamalvae* (Rossi, 1790)	LC	1
Apidae	*Tetralonianana* Morawitz, 1874	DD	2
Apidae	*Tetraloniastrigata* (Lepeletier, 1841)	DD	1
Apidae	*Xylocopairis* (Christ, 1791)	LC	1
Apidae	*Xylocopaviolacea* (Linnaeus, 1758)	LC	10
Colletidae	*Colletesalbomaculatus* (Lucas, 1848)	NT	5
Colletidae	*Colletessimilis* Schenck, 1853	LC	1
Colletidae	*Hylaeusangustatus* (Schenck, 1859)	LC	1
Colletidae	*Hylaeusbrachycephalus* (Morawitz, 1868)	DD	1
Colletidae	*Hylaeusbrevicornis* Nylander, 1852	LC	2
Colletidae	*Hylaeusclypearis* (Schenck, 1853)	LC	15
Colletidae	*Hylaeuscommunis* Nylander, 1852	LC	2
Colletidae	*Hylaeusconfusus* Nylander, 1852	LC	1
Colletidae	*Hylaeusgibbus* Saunders, 1850	LC	8
Colletidae	*Hylaeushyalinatus* Smith, 1842	LC	5
Colletidae	*Hylaeusimparilis* Förster, 1871	LC	3
Colletidae	*Hylaeusleptocephalus* Morawitz, 1870	LC	1
Colletidae	*Hylaeuslineolatus* (Schenck, 1861)	LC	1
Colletidae	*Hylaeuspfankuchi* (Alfken, 1919)	LC	1
Colletidae	*Hylaeuspictipes* Nylander, 1852	LC	9
Colletidae	*Hylaeuspictus* (Smith, 1853)	DD	12
Colletidae	*Hylaeuspunctatus* Brullé, 1832	LC	6
Colletidae	*Hylaeussignatus* Panzer, 1798	LC	1
Colletidae	*Hylaeusvariegatus* Fabricius, 1798	LC	5
Halictidae	*Halictusbrunnescens* (Eversmann, 1852)	DD	1
Halictidae	*Halictuscrenicornis* Bluthgen, 1923	DD	2
Halictidae	*Halictusfulvipes* (Klug in Germar, 1817)	LC	5
Halictidae	*Halictusmaculatus* Smith, 1848	LC	1
Halictidae	*Halictuspatellatus* Morawitz, 1873	LC	3
Halictidae	*Halictusscabiosae* (Rossi, 1790)	LC	11
Halictidae	*Halictussimplex* Bluthgen, 1923	LC	12
Halictidae	*Lasioglossumalbocinctum* (Lucas, 1849)	LC	4
Halictidae	*Lasioglossumbimaculatum* (Dours, 1872)	LC	31
Halictidae	*Lasioglossumbluethgeni* Ebmer, 1971	LC	2
Halictidae	*Lasioglossumglabriusculum* Morawitz, 1872	LC	4
Halictidae	*Lasioglossumgriseolum* Morawitz, 1872	LC	2
Halictidae	*Lasioglossumibericum* (Ebmer, 1975)	DD	1
Halictidae	*Lasioglossumlaticeps* (Schenck, 1868)	LC	2
Halictidae	*Lasioglossumlimbellum* Morawitz, 1872	DD	1
Halictidae	*Lasioglossumlineare* (Schenck, 1869)	DD	1
Halictidae	*Lasioglossummalachurum* Kirby, 1802	LC	28
Halictidae	*Lasioglossummediterraneum* Bluthgen, 1926	LC	10
Halictidae	*Lasioglossummesosclerum* Pérez, 1903	DD	3
Halictidae	*Lasioglossumminutissimum* Kirby, 1802	LC	2
Halictidae	Lasioglossum*morio* (Fabricius, 1793)	LC	6
Halictidae	Lasioglossum*nigripes* Lepeletier, 1841	LC	1
Halictidae	*Lasioglossumnitidulum* Fabricius, 1804	LC	16
Halictidae	*Lasioglossumpauperatum* Brullé, 1832	LC	1
Halictidae	*Lasioglossumpauxillum* (Schenck, 1853)	LC	3
Halictidae	*Lasioglossumpolitum* Schenck, 1853	LC	1
Halictidae	*Lasioglossumprasinum* Smith, 1848	NT	1
Halictidae	*Lasioglossumpygmaeum* (Schenck, 1853)	NT	18
Halictidae	*Lasioglossumsexnotatum* (Kirby, 1802)	NT	2
Halictidae	*Lasioglossumsoror* (Saunders, 1901)	EN	21
Halictidae	*Lasioglossumsubhirtum* (Lepeletier, 1841)	LC	3
Halictidae	*Lasioglossumtransitorium* (Schenck, 1869)	LC	96
Halictidae	*Lasioglossumvillosulum* Kirby, 1802	LC	1
Halictidae	*Lasioglossumzonulum* (Smith, 1848)	LC	2
Halictidae	*Nomiapisdiversipes* (Latreille, 1806)	LC	2
Halictidae	*Seladoniaconfusa* (Smith, 1853)	LC	1
Halictidae	*Seladoniagemmea* (Dours, 1872)	LC	5
Halictidae	*Seladoniapollinosa* (Sichel, 1860)	LC	1
Halictidae	*Seladoniasmaragdula* (Vachal, 1895)	LC	4
Halictidae	*Seladoniasubaurata* (Rossi, 1792)	LC	2
Halictidae	*Seladoniavestita* (Lepeletier, 1841)	LC	2
Halictidae	*Sphecodesephippius* (Linnaeus, 1767)	LC	1
Halictidae	*Sphecodespellucidus* Smith, 1845	LC	1
Halictidae	*Sphecodesruficrus* (Erichson, 1835)	LC	1
Megachilidae	*Anthidiellumstrigatum* Panzer, 1805	LC	9
Megachilidae	*Anthidiumdiadema* Latreille, 1809	DD	1
Megachilidae	*Anthidiumflorentinum* Fabricius, 1775	LC	12
Megachilidae	*Anthidiumloti* Perris, 1852	DD	4
Megachilidae	*Anthidiummanicatum* (Linnaeus, 1758)	LC	14
Megachilidae	*Anthidiumoblongatum* Immigur, 1806	LC	6
Megachilidae	*Chelostomadistinctum* Stoeckhert, 1929	LC	2
Megachilidae	*Chelostomaflorisomne* Linnaeus, 1758	LC	4
Megachilidae	*Chelostomarapunculi* (Lepeletier, 1841)	LC	1
Megachilidae	*Heriadescrenulata* Nylander, 1856	LC	14
Megachilidae	*Heriadestruncorum* Linnaeus, 1758	LC	16
Megachilidae	*Hoplitisacuticornis* (Dufour & Perris, 1840)	LC	1
Megachilidae	*Hoplitisadunca* Panzer, 1798	LC	32
Megachilidae	*Hoplitisanthocopoides* Schenck, 1853	LC	6
Megachilidae	*Hoplitisbenoisti* Alfken, 1935	LC	15
Megachilidae	*Hoplitisbisulca* (Gerstäcker, 1869)	LC	1
Megachilidae	*Hoplitisbrachypogon* (Pérez, 1880)	LC	1
Megachilidae	*Hoplitiscristatula* (Van der Zanden, 1990)	LC	11
Megachilidae	*Hoplitisleucomelana* (Kirby, 1802)	LC	1
Megachilidae	*Hoplitismocsaryi* (Friese, 1895)	LC	1
Megachilidae	*Hoplitisperezi* (Ferton, 1895)	LC	1
Megachilidae	*Megachileapicalis* (Spinola, 1808)	LC	3
Megachilidae	*Megachile*argentata Alfken, 1924	LC	6
Megachilidae	*Megachilecentuncularis* Linnaeus, 1758	LC	6
Megachilidae	*Megachileericetorum* Lepeletier, 1841	LC	3
Megachilidae	*Megachileflabellipes* Perez, 1895	DD	1
Megachilidae	*Megachilegiraudi* Gerstäcker, 1869	DD	1
Megachilidae	*Megachileleachella* Curtis, 1828	LC	1
Megachilidae	*Megachilemaritima* (Kirby, 1802)	DD	1
Megachilidae	*Megachilemelanopyga* Costa, 1863	LC	6
Megachilidae	*Megachileopacifrons* Pérez, 1897	DD	1
Megachilidae	*Megachileparietina* (Geoffroy in Fourcroy, 1785)	LC	12
Megachilidae	*Megachilepusilla* Pérez, 1884	DD	2
Megachilidae	*Megachilepyrenaica* Lepeletier, 1841	DD	4
Megachilidae	*Megachilerotundata* (Fabricius, 1793)	DD	4
Megachilidae	*Megachilesculpturalis* Smith, 1853		6
Megachilidae	*Megachilewillughbiella* Kirby, 1802	LC	3
Megachilidae	*Osmiaaurulenta* Panzer, 1799	LC	23
Megachilidae	*Osmiabicornis* Linnaeus, 1758	LC	34
Megachilidae	*Osmiabrevicornis* Fabricius, 1798	LC	5
Megachilidae	*Osmiacaerulescens* (Linnaeus, 1758)	LC	6
Megachilidae	*Osmiadimidiata* Morawitz, 1870	LC	1
Megachilidae	*Osmialatreillei* (Spinola, 1806)	LC	10
Megachilidae	*Osmialeaiana* (Kirby, 1802)	LC	4
Megachilidae	Osmia*ligurica* Morawitz, 1868	LC	3
Megachilidae	*Osmiamelanogaster* Spinola, 1807	LC	24
Megachilidae	*Osmiaminutula* (Pérez, 1896)	DD	1
Megachilidae	*Osmianasoproducta* Ferton, 1910	DD	8
Megachilidae	*Osmianiveata* (Fabricius, 1804)	LC	47
Megachilidae	*Osmiarufohirta* Latreille, 1811	LC	20
Megachilidae	*Osmiascutellaris* Morawitz, 1868	LC	10
Megachilidae	*Osmiasignata* Erichson, 1835	LC	3
Megachilidae	*Osmiasubmicans* Morawitz, 1870	LC	10
Megachilidae	*Osmiatricornis* Latreille, 1811	LC	56
Megachilidae	*Osmiaversicolor* Latreille, 1811	LC	9
Megachilidae	*Protosmiaminutula* (Pérez, 1896)	DD	3
Megachilidae	*Rhodanthidiuminfuscatum* (Erichson in Waltl, 1835)	DD	5
Megachilidae	*Rhodanthidiumseptemdentatum* Latreille, 1809	DD	171
Megachilidae	*Rhodanthidiumsticticum* (Fabricius, 1787)	DD	162
Megachilidae	*Stelisbreviuscula* Nylander, 1848	LC	1
Megachilidae	*Stelissignata* (Latreille, 1809)	LC	2
Melittidae	*Dasypodaargentata* Panzer, 1809	NT	1
Melittidae	*Dasypodahirtipes* (Fabricius, 1793)	LC	1
**Total**			**2181**
